# HIV and fungal priority pathogens

**DOI:** 10.1016/S2352-3018(23)00174-1

**Published:** 2023-10-09

**Authors:** Hatim Sati, Ana Alastruey-Izquierdo, John Perfect, Nelesh P. Govender, Tom Harrison, Tom Chiller, Tania Sorrell, Felix Bongomin, Rita Oladele, Arunaloke Chakrabarti, Retno Wahyuningsih, Arnaldo Lopes Colombo, Juan Luis Rodriguez Tudela, Chris Beyrer, Nathan Ford

**Affiliations:** 1 Antimicrobial Resistance Division, WHO, Geneva, Switzerland; 2 Mycology Reference Laboratory, National Center for Microbiology, Instituto de Salud Carlos III, 28220 Madrid, Spain; 3 Global Action for Fungal Infections, 1208 Geneva, Switzerland; 4 Division of Infectious Diseases, Department of Medicine, Duke University School of Medicine, Durham, North Carolina, USA; 5 National Institute for Communicable Diseases, a Division of the National Health Laboratory Service, School of Pathology, University of the Witwatersrand, Johannesburg, South Africa; 6 Centre for Global Health, Institute of Infection and Immunity, St George’s University of London, London, United Kingdom; 7 Centers for Disease Control and Prevention (CDC), Atlanta, Georgia, USA; 8 Sydney Infectious Disease Institute, University of Sydney, Sydney, NSW 2145, Australia; 9 Department of Medical Microbiology and Immunology, Faculty of Medicine, Gulu University, Gulu, Uganda; 10 Department of Medical Microbiology and Parasitology, Faculty of Basic Medical Sciences, College of Medicine, University of Lagos, Lagos, Nigeria; 11 Doodhdhari Burfani Hospital and Research Institute, Haridwar, India; 12 Universitas Indonesia and Universitas Kristen Indonesia, Indonesia; 13 Department of Medicine, Division of Infectious Diseases, Federal University of São Paulo, Brazil; 14 Duke Global Health Institute, Duke University, Durham, North Carolina, USA; 15 Global HIV, Hepatitis and STI Programmes, World Health Organization, Geneva, Switzerland; 16 Centre for Infectious Disease and Epidemiology and Research, School of Public Health and Family Medicine

## Abstract

The burden of invasive fungal infections associated with opportunistic fungal pathogens is a persistent challenge, particularly among people with advanced HIV disease. In October, 2022, WHO published the Fungal Priority Pathogens List (FPPL)—the first global effort to systematically prioritise fungal pathogens. Of the 19 pathogens in the WHO FPPL, four opportunistic pathogens in particular cause invasive diseases in people living with HIV: *Cryptococcus neoformans, Histoplasma* spp*, Pneumocystis jirovecii*, and *Talaromyces marneffei*. These four fungal pathogens are major causes of illness and death in people with advanced HIV and overwhelmingly affect those in low-income and middle-income countries. Access to diagnostics, improved surveillance, targeted support for innovation, and an enhanced public health focus on these diseases are needed in the effort to reduce HIV-associated deaths.

## Introduction

The burden of disease associated with invasive fungal infections is rising due to growth of immunocompromised populations (including people with advanced HIV), new viral comorbidities (eg, SARS-CoV-2 and influenza), and improved diagnostic tests allowing easier identification. However, there is a paucity of data on the distribution of fungal disease globally, with no systematic assessment or reporting of infections. Therefore, fungal infections receive insufficient recognition from national and global agencies, and their prevention, control, and treatment are inadequately funded.

In October, 2022, WHO released the Fungal Priority Pathogens List (FPPL)—the first global effort to systematically prioritise fungal pathogens. The FPPL was developed after expert review of both the public health importance and diagnostic and therapeutic development needs associated with each pathogen.^1^ The prioritisation process was led by WHO in consultation with a group of experts in fungal diseases and was informed by a series of systematic reviews, which provided updated estimates of the disease burden and effect of fungal infections on human health. The focus was on pathogens that cause invasive fungal diseases for which there is insufficient access to effective, rapid, and early diagnosis and treatment, and the efficacy of existing preventives and therapeutics are threatened by the rise of antifungal resistance. 19 fungal pathogens were assessed against ten criteria: mortality, annual incidence, global distribution, changes in incidence and prevalence patterns in the last 10 years, inpatient care, complications from disease, antifungal resistance, preventability, and access to diagnostic tests and evidence-based treatments.

Of the 19 pathogens in the WHO FPPL, four opportunistic pathogens cause invasive diseases in people living with HIV: *Cryptococcus neoformans, Histoplasma* spp*, Pneumocystis jirovecii*, and *Talaromyces marneffei* (previously known as *Penicillium marneffei*). In this Viewpoint we summarise gaps in prevention, diagnosis, and treatment of infections caused by these priority pathogens and opportunities for innovation and improved access to diagnostics and therapeutics (figure).

### Cryptococcus spp

Cryptococcal disease is one of the most common opportunistic infections in people living with advanced HIV and is a major contributor to morbidity and mortality. The dominant pathogenic species is *Cryptococcus neoformans*, which is a key priority pathogen ranked number 1 according to the WHO FPPL. *C gattii* infections are less common and were ranked as medium priority; however, the resulting disease in people with advanced HIV is indistinguishable from those caused by *C neoformans*.^2^

Globally, cryptococcosis accounts for one in five AIDS-related deaths. An updated review^3,4^ of the burden of cryptococcal disease estimated that there were 179 000 cases of cryptococcal infection (antigenaamia) and 152 000 cases of cryptococcal meningitis globally in 2020, which resulted in 112 000 cryptococcal-related deaths that year. Despite widespread use of antiretroviral treatment, incidence of cryptococcosis remains high. A study^5^ from sub-Saharan Africa found that over half of individuals diagnosed with the first episode of cryptococcal meningitis were antiretroviral drug experienced. WHO recommends screening for cryptococcal antigenaemia in adults and adolescents with a CD4 cell count of less than 200 cells per μL.^6^ Accurate and affordable point-of-care cryptococcal antigen screening with lateral flow assays helps determine the need for pre-emptive antifungal therapy among those with antigenaemia; this intervention can reduce the incidence of meningitis and cryptococcosis-related mortality.^7^ Implementation of this life-saving recommendation is hampered by uneven access to this screening tool globally and by inadequate follow-up of individuals with antigenaemia for timely initiation and continuation of pre-emptive fluconazole.

Advances in the management of cryptococcal meningitis have simplified treatment for all, including for people living with HIV. Guidelines released by WHO in 2022 strongly recommend a single high dose of liposomal amphotericin B, followed by 2 weeks of flucytosine and fluconazole as the preferred induction treatment.^6^ The regimen containing a single dose of liposomal amphotericin B has a lower risk of side-effects than longer courses, reducing toxicity monitoring needs, which might allow for faster hospital discharge.^8,9^ This regimen is only marginally more expensive than the regimen previously recommended by WHO (1-week daily doses of amphotericin B deoxycholate), which was also associated with serious off-target toxic effects (including nephrotoxicity and anaemia).^10^

The persistently high mortality from cryptococcal meningitis among people living with HIV is partly because of delayed diagnosis, which results from the non-specific nature the initial symptoms (ie, headache), poor access to rapid diagnostic assays, and the need for lumbar puncture to establish the diagnosis and then to manage raised intracranial pressure. Access to liposomal amphotericin B and companion drugs is another major challenge,^11^ due to multiple market failures, including high cost and limited national drug registration (partly as a result of small capacity and poor coordination of drug regulatory authorities in many countries), low demand from countries, poor demand forecasting estimates, and few suppliers. More clinical experience is needed with the single high-dose liposomal amphotericin B regimen in countries where this treatment is available; currently the combination of liposomal amphotericin B plus flucytosine is usually still given for a 2-week induction period in countries with sufficient resources.^12^

### Histoplasma spp

Histoplasmosis is another global opportunistic infection, and one of the most common among people with advanced HIV disease in Latin America. *Histoplasma* spp lives as a mould in the environment. Most people who inhale the spores of *Histoplasma* spp are asymptomatic or develop symptoms not requiring medical attention. However, people living with HIV and other immunocompromised patients, such as people with cancer and organ transplant recipients, can develop severe disseminated forms of histoplasmosis, and mortality can be high. *Histoplasma* spp are ranked as a high priority in the WHO FPPL.

The most frequent clinical presentation in people living with HIV is progressive disseminated histoplasmosis. Symptoms are non-specific and often indistinguishable from other infectious diseases (eg, disseminated tuberculosis), which complicates management. In 2020, WHO and the Pan American Health Organization released standard guidelines for the diagnosis, treatment, and management of disseminated histoplasmosis in people living with HIV.^13^

Histoplasmosis is highly endemic in some parts of the Americas, where it causes 15 600 new infections and 4500 deaths among people living with HIV each year. Infections are also reported across Asia and Africa, but the disease burden in these regions is unknown, in part due to inadequate diagnostic capacity and surveillance. Access to traditional diagnostic methods—cell culture and histopathology—are restricted by the need for complex laboratory infrastructure, trained staff, and long turnaround times affecting treatment outcomes. Antigen detection methods have improved diagnostic accuracy, but these technologies are not yet widely available or optimised for accurate diagnosis in the lateral flow assay format. There is generally a low clinical index of suspicion, which should be improved through training of health-care providers.

Access to appropriate antifungal therapies, including liposomal amphotericin B and itraconazole, is inadequate. Fluconazole has inferior therapeutic efficacy compared with itraconazole, and development of fluconazole resistance^14^ is another important limitation of this drug. Licensing of liposomal amphotericin B and itraconazole is encouraged so that skilled clinicians can use them where most needed. A phase 2 multicentre randomised trial^15^ found that a single high dose of liposomal amphotericin B is safe, which indicates an opportunity to shorten and simplify treatment and this is under evaluation in a phase 3 trial.

### P jirovecii

Pneumocystis pneumonia is a leading cause of mortality in hospitalised adults and children living with HIV. *P jirovecii* is transmitted from person to person through the air. The main ways to prevent and treat this infection among people living with HIV, according to WHO and most national guidelines, are use of co-trimoxazole and rapid early initiation of antiretroviral therapy.^16^

Diagnosis of pneumocystis pneumonia has traditionally relied upon clinical symptoms, radiographic findings, and microscopy because *P jirovecii* cannot be cultured. Non-culture-based diagnostics on sputum or bronchoalveolar lavage, such as PCR, have entered clinical use, but there is often inadequate infrastructure for these assays in resource-limited settings.^17^ A review of published studies found a disease prevalence of 19% among symptomatic adults with HIV in Africa, with little change despite increased access to prophylaxis regimens.^18^

*P. jirovecii* was ranked as medium priority in the WHO FPPL, considering the high availability and affordability of prophylaxis and treatment, and moderate availability of conventional diagnostics. Nevertheless, accurate and cost-effective point-of-care diagnostic options for *P jirovecii* infections are needed. Antigen testing, which is effective for diagnosis of other fungal infections, is a promising approach in development; several other diagnostic techniques show promise in research settings, but their applicability in resource-limited settings remains unclear.^17^ Existing antifungal therapies are associated with important toxic effects, drug resistance, and access challenges, and a number of alternative therapeutic options to overcome these challenges can be considered.^19^

### T marneffei

Talaromycosis is one of the most common invasive fungal diseases in people with advanced HIV disease in southeast Asia. *T marneffei* is endemic in higher altitude regions of southeast Asia and parts of China, India, and Indonesia, with a reported prevalence of more than 10% in some regions.^20^ The fungus is found in the environment and causes lung disease with multiorgan haematogenous dissemination in humans after inhalation of spores. The clinical manifestations commonly include skin lesions, which can be stigmatising for people who live in endemic areas. Like most fungal infections acquired from the environment, there is no human-to-human transmission.

Invasive talaromycosis affects people living with HIV and other immunocompromised patients, and in endemic areas it can be responsible for up to 15% of HIV-related hospital admissions. Talaromycosis mortality rates of up to 30% have been reported, which can be reduced with amphotericin-B induction therapy.^21-23^ Diagnosis is presumptive, based on microscopy; although PCR-based assays are available, their sensitivity is low. Antigen tests, including rapid point-of-care tests, are in development, and if sufficiently accurate, they could be used for screening of early talaromycosis in patients with advanced HIV disease.^24^ Given its restricted geographical distribution, *T marneffei* is considered to be of medium priority by the WHO FPPL.

## Other invasive fungal pathogens

People with advanced HIV disease are at risk of illness and death from a wide range of infectious diseases. *Candida* spp, *Paracoccidioides* spp, *Coccidioides* spp, and *Aspergillus fumigatus* most commonly cause severe disease in other patient populations, but they are also important opportunistic pathogens in people living with HIV.^25^ Severe disease in people living with HIV caused by most of the pathogens on the FPPL is frequently reported. In Brazil, mortality of 73% was reported in a retrospective review of outcomes of 11 severely immunocompromised people with HIV and invasive aspergillosis.^26^ In South Africa, people with HIV admitted to hospital with candidaemia had almost twice the risk of mortality compared with HIV-seronegative patients.^27^

## WHO FPPL and priority areas for action for HIV

The four fungal diseases highlighted in this Viewpoint are among the major causes of infections and death in people with advanced HIV, and overwhelmingly affect people in low-income and middle-income countries. Unfortunately, there is little data regarding the complications and sequelae of infections with these and other fungal pathogens. Also, clinical questions on including optimal diagnostic approach, preventive and treatment regimens, and duration of inpatient care remain unanswered. Many laboratories worldwide do not have the expertise of specialised mycology laboratories, where microscopic examination, cell culture, and morphological identification have long been the primary methods for diagnosis of fungal infections. A survey of diagnostic capacity for invasive fungal pathogens across 48 countries in Africa between 2020 and 2022 found an insufficient number of diagnostic tools leading to under-reporting of disease burden, which in turn leads to insufficient resource allocation for diagnosis and treatment.^28^ Similarly, a 2018 survey^29^ of mycological diagnostic and therapeutic capabilities in 24 countries in Latin America and the Caribbean concluded that there is an urgent need to improve diagnostic capacity and access to treatment. A 2022 survey^30^ across 40 countries in the Asia-Pacific region found that resources for diagnosing and access to treatment for invasive fungal pathogens varied across the region; the survey concluded that regional cooperation could help overcome multiple limitations.

There is a need to use new point-of-care diagnostics at all levels of HIV care as affordable and accurate diagnostic tests support earlier initiation of therapeutic strategies leading to improved treatment outcomes. Improvement of surveillance, targeted support for innovation, and enhanced public health interventions are some of the other priorities for reducing HIV-associated illness and death.

Progress in laboratory surveillance of *C neoformans, Histoplasma* spp*, P jirovecii*, and *Ts marneffei* infections will depend on access to laboratories with mycology diagnostic capacity, particularly in settings with a high HIV prevalence; development and adoption of cheap, accurate lateral flow assays in all settings should be the goal. Some lateral flow assays are in development, but cryptococcal antigen assays are commercially available through several manufacturers (although their performance varies). Mycology laboratories can be added to health-care facilities, where people living with HIV receive care, are diagnosed, and surveillance programmes are already in place. This approach takes advantage of existing health-care infrastructure to enhance the diagnosis and treatment of opportunistic fungal infections. Efforts have been made (in particular, the development of alternative diagnostic methods) to overcome the challenges that have prevented diagnosis of life-threatening invasive fungal diseases due to poor access to mycology laboratories. Precedence for this approach was established with another slowly replicating microorganism—Mycobacterium tuberculosis— the handling of which also requires biosafety level 3 facilities. Lateral flow assay devices and commercial PCR techniques have made diagnostic procedures accessible to laboratories without specialised tuberculosis or mycology expertise. In areas where microbiology facilities are available, it is essential to use techniques that provide rapid results. Virologists routinely use PCR, lateral flow assays, ELISA sequencing, and other techniques; they possess the necessary technical expertise to extend diagnostic capabilities to other microbiology specialties, including mycology.

Linkage of laboratory and clinical data can help to estimate the burden of fungal infections and antifungal resistance in people living with HIV. Such integrated data are scarce, especially in low-income settings. Improved clinical screening of populations at risk of fungal infections will depend on the level of training of the health-care worker and will be supported by community knowledge and education about clinical presentations and risk factors for these infections among people living with HIV. Affordable access to diagnostic tools at the point of care is essential for optimal patient care and screening of at-risk populations for acquisition of accurate surveillance data. Commitment made by donor institutions, implementing partners, and governments in individual countries is needed to facilitate robust data collection and reporting.

Given the high mortality rates associated with invasive fungal diseases in people with HIV treated with many of the available therapies, novel antifungal agents and improved regimens are urgently needed. Further research and innovation should optimise the way current antifungals and diagnostics are used, such as strategies to improve availability of therapeutic antifungal monitoring and to optimise combination therapies that prevent drug resistance emergence and increase efficacy. Investment in the development of novel antifungals and affordable diagnostic tools for invasive fungal pathogens affecting people living with HIV is essential and can be leveraged to diagnose and treat these mycoses in other growing immunosuppressed patient populations.

Finally, education on the importance of fungal pathogens among people living with HIV should be expanded. Health-care workers in many settings are unfamiliar with fungal infections, resulting in low clinical suspicion, misdiagnosis, incorrect or delayed treatment, and poor outcomes. Fungal infections need to be included in the early and ongoing medical and public health training, and specifically integrated into HIV care training.^31^

Advanced HIV disease is receiving increased attention from major donors including the Global Fund to Fight AIDS, Tuberculosis and Malaria, the US Presidents’ Emergency Plan for AIDS Relief, and Unitaid. Important initiatives have been launched in the last 5 years, including a drive to End Cryptococcal Deaths in HIV by 2030.^32^ There is a need to increase the recognition of and focus on fungal diseases, with the goal to support development and to scale up access to preventives, diagnostics, and treatments for infections caused by these pathogens. Early and effective therapies can make a difference in fungal infection outcome, and improved diagnostics and treatments are needed.

## Figures and Tables

**Figure:**
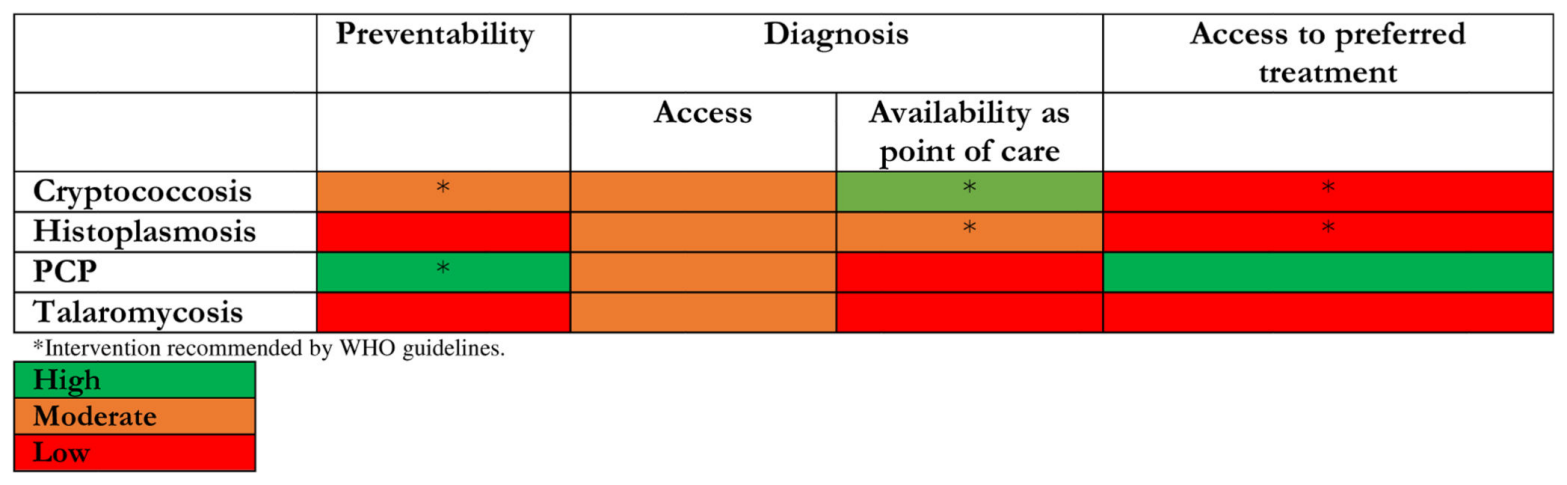
Prevention, diagnosis, and treatment of HIV-associated infections caused by priority fungal pathogens in low-income and middle-income countries
